# Clustered multistate models with observation level random effects, mover–stayer effects and dynamic covariates: modelling transition intensities and sojourn times in a study of psoriatic arthritis

**DOI:** 10.1111/rssc.12235

**Published:** 2017-07-25

**Authors:** Sean Yiu, Vernon T. Farewell, Brian D. M. Tom

**Affiliations:** ^1^ University of Cambridge UK

**Keywords:** Clustered processes, Jump probabilities, Mover–stayer model, Multistate model, Psoriatic arthritis, Sojourn times

## Abstract

In psoriatic arthritis, it is important to understand the joint activity (represented by swelling and pain) and damage processes because both are related to severe physical disability. The paper aims to provide a comprehensive investigation into both processes occurring over time, in particular their relationship, by specifying a joint multistate model at the individual hand joint level, which also accounts for many of their important features. As there are multiple hand joints, such an analysis will be based on the use of clustered multistate models. Here we consider an observation level random‐effects structure with dynamic covariates and allow for the possibility that a subpopulation of patients is at minimal risk of damage. Such an analysis is found to provide further understanding of the activity–damage relationship beyond that provided by previous analyses. Consideration is also given to the modelling of mean sojourn times and jump probabilities. In particular, a novel model parameterization which allows easily interpretable covariate effects to act on these quantities is proposed.

## 1 Introduction

In psoriatic arthritis, manifestations of the disease typically result in joints becoming swollen and/or painful (active joints), which are reversible through treatment or management strategies or spontaneously, and may lead to permanent joint damage. The interplay between disease activity (as measured by activity in the joint) and damage is believed to be of a causal nature with a previous investigation performed by O’Keeffe *et al*. (2011) providing an extensive discussion on the topic. In that analysis, among others, individual joint level three‐state models consisting of a not‐active and not‐damaged state, active and not‐damaged state and an absorbing damaged state were proposed and produced strong evidence of a greatly increased transition rate to damage when a joint is active (compared with a joint being not active). The three‐state models were fitted under a working independence assumption (the three‐state processes are independent within an individual) with a robust covariance matrix used to adjust standard errors (Lee and Kim, 1998). The purpose of this paper is to extend the current modelling framework so that greater confidence with regard to the association between activity and damage can be achieved, and also to inform on other important clinical questions.

From a statistical point of view, it is important to adjust for observed and, where possible, unobserved characteristics which are believed to be strongly related to the processes of interest, i.e. confounder variables. An analysis that does not may produce spurious associations between included covariates and the outcome. Therefore, as extensions to O’Keeffe *et al*. (2011), dynamic covariates (covariates which describe important aspects of previous developments of the process) are included to allow current transition intensities to depend on previous history (relaxing the Markov assumption), random effects are introduced into the transition intensities to account for unobserved heterogeneity and provide a more efficient estimation procedure, and a mover–stayer model (Frydman, 1984) is considered to allow for the possibility that some patients (stayers) have no propensity to develop damaged joints. Whereas much research has focused on the effect of disease activity on joint damage, no research has yet considered the reverse association (i.e. the effect of damage on activity). Specifically, it is of interest to investigate whether the disease activity process changes with onset of damage, and how if so. To inform on the possible association, the absorbing damaged state is further subdivided into an active and damaged state and a not‐active and damaged state, thereby allowing the disease activity process to be modelled even after a joint has become damaged. The resulting model then utilizes the entire data set, as opposed to previously where the disease activity process was stopped once a joint had become damaged. By considering a mover–stayer model, it is also possible to investigate whether the activity process is different between movers (those who have the propensity to develop damaged joints) and stayers, and this will also contribute new knowledge towards the relationship between damage and activity.

Clustered progressive multistate models constructed using random effects have previously been proposed in the panel data literature. See, for example, Cook *et al*. (2004), O’Keeffe *et al*. (2012) and Sutradhar and Cook (2008). In our context, these models introduce time invariant, possibly multivariate random effects at the patient level to account for the correlation between joints from the same patient, time invariant unobserved heterogeneity and relaxation of the Markov assumption. A novel feature of our work is the use of observation level multivariate random effects in clustered non‐progressive multistate models to account for correlation and time varying unobserved heterogeneity and the introduction of dynamic covariates to relax the Markov assumption explicitly. The proposing of this random‐effects structure was motivated by the extensive lengths of follow‐up and the possibly non‐predictable changes in unobserved heterogeneity due to treatment or management strategies employed by the clinic and the spontaneous nature of joint activity. Such observations are less likely to result in unobserved heterogeneity being time invariant or time varying but deterministic (which is enforced by patient level random effects). Along with generalized estimating equations, copulas (Diao and Cook, 2014) and expanded state space models (Tom and Farewell, 2011) have also been proposed to handle clustering. Although there are considerable advantages to such models, they are particularly difficult to formulate and implement when more than two intermittently observed non‐progressive multistate processes are of interest.

The natural multistate modelling parameterization allows covariates to act on transition intensities in a proportional hazards framework. Therefore easily interpretable covariate effects on these transition intensities can be obtained. Another natural way to view a multistate process is in terms of its sojourn times (the time spent in a state before a transition occurs) and jump probabilities (the probability of transitioning to a state given that a transition occurs). If these quantities are of interest, a model parameterization which allows easily interpretable covariate effects to act on these quantities would be useful, especially as current parameterizations may not enable such interpretation. We consider this issue to motivate a modification of the original three‐state model.

The next section introduces the psoriatic arthritis data on which this analysis is based.

## 2 Psoriatic arthritis data

**Figure 1:**
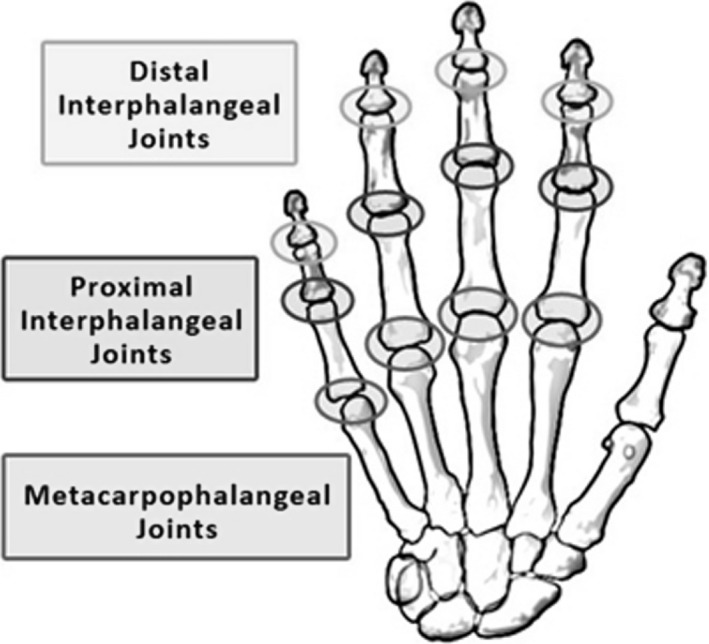
Diagram of the type of joints in each hand: this investigation focuses on 14 joints in each hand consisting of the distal interphalangeal, proximal interphalangeal and metacarpophalangeal joints in each finger and the proximal interphalangeal and metacarpophalangeal joints in the thumb (the figure was obtained from the arthritis fact sheet on the Georgia Tech Web page usability.gtri.gatech.edu)

Psoriatic arthritis is an inflammatory arthritis that is associated with the skin condition psoriasis. At the University of Toronto psoriatic arthritis clinic, over 1000 patients have been followed up longitudinally since it began in 1978 with clinic visits scheduled 6–12 months apart. In particular, at these visits, the active and damaged joint counts are recorded at the individual joint level, among other measurements, and therefore permit statistical modelling at this level of detail. In this investigation, focus will be on the 28 hand joints (14 joints in each hand; see Fig. 1 for more details), which can result in severe physical disability if active and/or damaged. Furthermore, this investigation is based on 743 patients who entered the clinic with no damage in either hand, so that patients are more comparable in their initial state of disease progression, and had more than two clinic visits. A dynamic covariate which requires previous observations will be constructed in the next section. Of this subset of patients, 69% (514 of 743 patients) had no damage at the end of their follow‐up, which motivates consideration of a stayer population. The mean follow‐up time was 10 years and 8 months with an interquartile range of 11 years and 6 months. The mean and median number of clinic visits were 12.7 and 8 respectively, and this ranged from 2 to 57. The mean and median intervisit times were 10 and 6 months, with a standard deviation of 1 year and 3 months. At clinic entry, the mean age at onset of arthritis was 36 years and 8 months with a standard deviation of 13 years and 4 months, whereas the mean duration of arthritis was 5 years and 2 months with a standard deviation of 7 years and 2 months. Furthermore, 55% of patients were male and 45% female.

In total, there were 264208 observed transitions over all hand joints in the data. The observed transition matrix is

A¯D¯AD¯A¯DADA¯D¯(21797611008623158)AD¯122508599200169A¯D0010935680AD00882728

where $\overset{¯}{A}$ and *A* denote the absence and presence of activity in the joint respectively, and $\overset{¯}{D}$ and *D* denote whether the joint has been clinically assessed as not damaged and damaged respectively.

The next section describes a six‐state model which will be useful for jointly investigating the activity and damage processes.

## 3 Six‐state model for transition intensities

**Figure 2:**
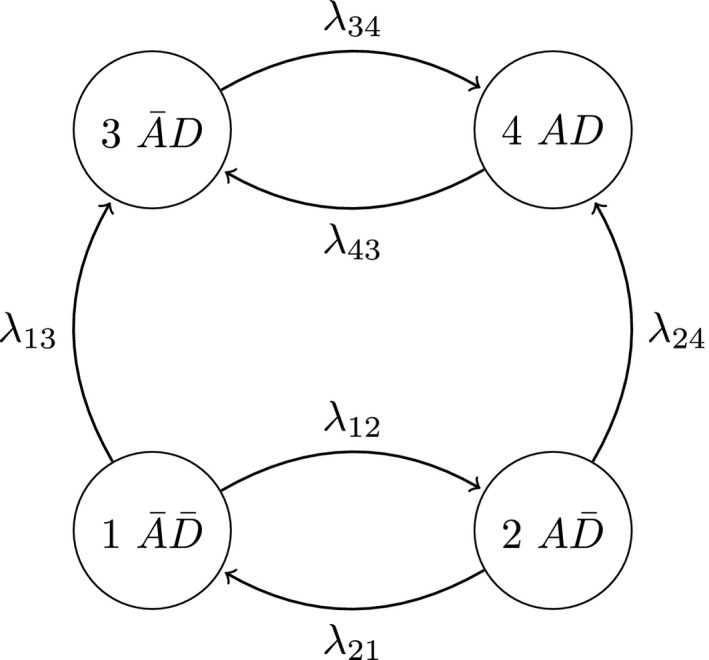
Multistate model describing the disease activity and damage processes jointly for movers

Multistate models provide a convenient framework when the evolution of a stochastic process is of interest (Commenges, 1999; Anderson, 2002). This investigation demonstrates their use for the joint analysis of the disease activity and damage processes occurring in each individual hand joint. Specifically, consider the following four‐state representation that is depicted in Fig. 2.

**Figure 3:**
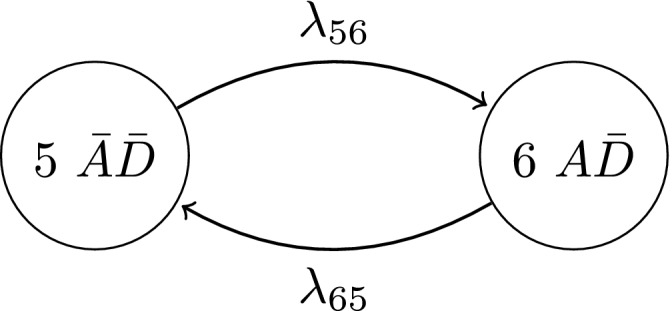
Multistate model describing the activity process for stayers

The process characteristics, particularly the reversibility of activity and permanent nature of damage, are reflected in the non‐zero transition intensities which describe the instantaneous rate of transitioning between states. It is implicit that this representation describes the possible transitions of movers since *λ*_13_ and *λ*_24_>0. If, however, a stayer population exists with regard to developing damaged hand joints, their disease activity process can be described by the multistate diagram in Fig. 3.

Let $\lambda_{rsij}^{l}$ denote the transition intensity from state *r* to *s* for the *l*th joint of the *i*th patient at the *j*th clinic visit (at each clinic visit all 28 hand joints are observed). Initially to investigate specific clinical aspects described in Section 1 (and to formulate a more parsimonious model), the transition intensities are parameterized as follows:

(1) $$\left.\begin{array}{cc} & \lambda_{34ij}^{l}=\lambda_{12ij}^{l}\;\text{exp}\left(\beta_{\mathrm{Damaged}}^{\overset{¯}{A}A}\right),\\ & \lambda_{43ij}^{l}=\lambda_{21ij}^{l}\;\text{exp}\left(\beta_{\mathrm{Damaged}}^{A\overset{¯}{A}}\right),\\ & \lambda_{24ij}^{l}=\lambda_{13ij}^{l}\;\text{exp}\left(\beta_{\mathrm{Active}}^{\overset{¯}{D}D}\right),\\ & \lambda_{56ij}^{l}=\lambda_{12ij}^{l}\;\text{exp}\left(\beta_{\mathrm{Stayer}}^{\overset{¯}{A}A}\right),\\ & \lambda_{65ij}^{l}=\lambda_{21ij}^{l}\;\text{exp}\left(\beta_{\mathrm{Stayer}}^{A\overset{¯}{A}}\right). \end{array}\right\}$$

**Figure 4:**
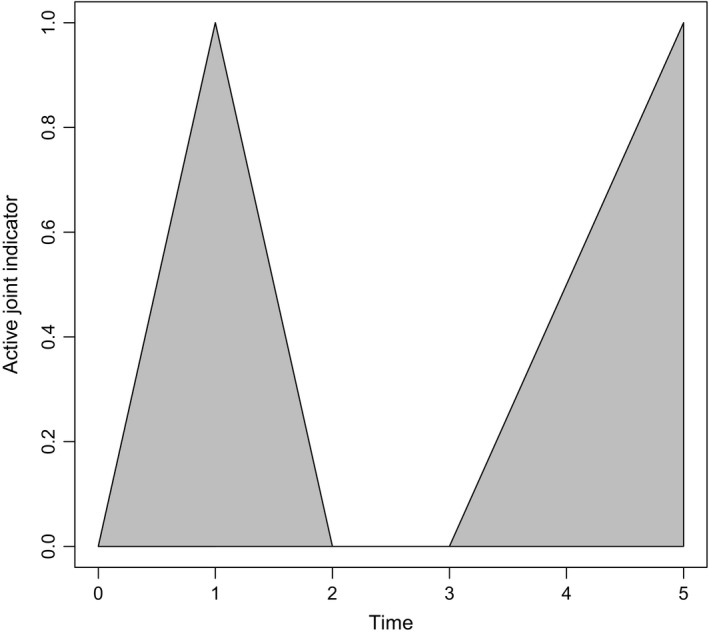
Let *x*(*t*) be realized values of *X*(*t*), a binary stochastic process describing the activity process of a joint: specifically at time *t*,* x*(*t*)=1 corresponds to the joint being active and *x*(*t*)=0 corresponds to the joint being not active; AMA(*t*) is then calculated as $\left(1/t\right)\int_{0}^{t}x\left(s\right)\;ds$, thus resulting in a bounded measure between [0,1]; as *x*(*t*) is intermittently observed and therefore the true path of *x*(*t*) is not known, $\int_{0}^{t}x\left(s\right)\;ds$ is approximated as the area under the linearly interpolated observations of *x*(*t*); for example, if a joint was observed at *t*=(0,1,2,3,5) such that *x*(*t*)=(0,1,0,0,1) respectively, then AMA(5) is approximated as the shaded area divided by 5, i.e. hence 0.4

Thus regression coefficients are used to provide a simple representation of the main effect of damage and mover–stayer status on activity transitions as well as the main effect of activity on the damage transition. We note that more complex models involving interaction effects can be developed for estimating differential effects across various subgroups of patients. Furthermore, we let

(2) $$\left.\begin{array}{cc} & \lambda_{12ij}^{l}=\lambda_{0}^{\overset{¯}{A}A}\;\text{exp}\left(\beta^{\overset{¯}{A}A}z_{ij}^{l}+u_{ij}\right),\\ & \lambda_{21ij}^{l}=\lambda_{0}^{A\overset{¯}{A}}\;\text{exp}\left(\beta^{A\overset{¯}{A}}z_{ij}^{l}+\alpha u_{ij}\right),\\ & \lambda_{13ij}^{l}=\lambda_{0}^{\overset{¯}{D}D}\;\text{exp}\left(\beta^{\overset{¯}{D}D}z_{ij}^{l}+v_{ij}\right), \end{array}\right\}$$

where $\lambda_{0}^{\overset{¯}{A}A}$, $\lambda_{0}^{A\overset{¯}{A}}$ and $\lambda_{0}^{\overset{¯}{D}D}$ are constant baseline intensities, $\beta^{\overset{¯}{A}A}$, $\beta^{A\overset{¯}{A}}$ and $\beta^{\overset{¯}{D}D}$ are vectors of regression coefficients, $z_{ij}^{l}$ is a vector of covariates that are associated with the *l*th joint from the *i*th patient at the *j*th clinic visit, and *u*_*ij*_ and *v*_*ij*_ are realizations of zero‐mean bivariate normal observation level random effects. Here α∈R is an unknown parameter to be estimated which allows *u*_*ij*_ to act differently across the different transition intensities associated with the activity process. Although not formally stated, we include time‐dependent dynamic covariates in $z_{ij}^{l}$ to relax the Markov assumption. Specifically, the observed history of the activity process is summarized through a joint‐specific covariate denoted as the adjusted mean activity AMA (Ibañez *et al*. (2003); Fig. 4 provides a description), whereas a patient's state of disease progression is reflected through the current number of damaged joints attained. On average, AMA was calculated as 0.093 with a standard deviation of 0.19 in our data. Given these dynamic covariates, current transition intensities from the multistate process are then assumed independent of previous process history, i.e. the Markov assumption. The random effects are assumed independent across time (with respect to *j*) and can be seen as accounting for unobserved heterogeneity not due to previous process history (where adjustments to unobserved heterogeneity related to previous history are provided through the dynamic covariates), which is still unaccounted for in the model. It is worth noting that the explicitly specified regression coefficients in expressions (1) and (2) correspond to covariates with different modelling assumptions. The covariates in $z_{ij}^{l}$ are assumed to remain constant between clinic visits and therefore are relevant when this is true (time invariant covariates) or a reasonable approximation (e.g. when the covariate process is unlikely to be highly fluctuating between clinic visits). For simplicity, such covariates are also usually included if understanding the relationship between these covariates and the outcome is not of primary interest but some form of adjustment for these covariates is necessary. In contrast, the regression coefficients *β*_Damaged_ and *β*_Active_ are describing the effect of a binary variable representing a joint being damaged and active respectively while reflecting the stochastic nature of these processes and therefore provide more realistic measures of association. This is especially useful because these are the clinical aspects of primary interest. The regression coefficient *β*_Stayer_ is similar in nature to *α* as it describes the effect of a partially observable binary variable (stayer = 1 and mover = 0); it can only be known that patients with damage are movers and that patients with no damage are either movers or stayers.

The motivation behind the use of observation level random effects arose from the potential need to allow for non‐predictable changes in unobserved heterogeneity (Unkel *et al*., 2014) with regard to the damage and activity processes. The current methodology in the literature primarily uses patient level random effects. This forces unobserved heterogeneity to be time invariant and we assess this assumption in our context by fitting this model (i.e. {*U*_*ij*_,*V*_*ij*_}={*U*_*i*_,*V*_*i*_} ∀ *j*) in addition to the model proposed. Likelihood values can then be used to compare informally the usefulness of the modelling framework proposed (models are non‐nested but contain the same number of parameters; hence the same penalty terms are obtained if information criteria such as the Akaike information criterion are used). In general, the estimability of random‐effects models, particularly variance components, will be driven by the random‐effects structure and the variability of the data. The observation level random‐effects structure incorporates fewer shared random effects between transition intensities than does its patient level counterpart. As a consequence, there will be less variability between transition intensities containing the same random effect, thus making it more difficult to estimate the random‐effect variance when using the observation level random‐effects structure. This would especially be so when a single multistate process is of interest, where substantial heterogeneity in the data (which could be generated by constraining transition intensities) will probably be needed for observation level random‐effects models to be estimable, whereas patient level random‐effects models are likely to require considerably less heterogeneity (Satten (1999) and Cook (1999) considered a single multistate process with patient level random effects). In a clustered multistate process framework, heterogeneity is also generated through the differences across several multistate processes; thus the observation level random‐effects structure is far more likely to be estimable, especially as the number of processes increases.

### 3.1 Maximum likelihood estimation

The model proposed is fitted by constructing and then maximizing the marginal likelihood. Let $X_{i}^{l}\left(t_{ij}\right)$ denote the six‐state process for the *l*th joint from the *i*th patient at time *t*_*ij*_, where $\left\{t_{i1},\ldots ,t_{im_{i}}\right\}$ denotes the times of the *j*th clinic visit from the *i*th patient. Let *C*_*i*_ be a partially observable binary variable such that *C*_*i*_=1 with probability 1−*π*_*i*_ if patient *i* is a mover (transitions are governed by the four‐state model in Fig. 1), and *C*_*i*_=0 with probability *π*_*i*_ otherwise (transitions are governed by the two‐state model in Fig. 2). Under the assumption that the conditional process (conditional on the piecewise constant dynamic covariates, {*U*_*ij*_=*u*_*ij*_,*V*_*ij*_=*v*_*ij*_} ∀ *j* and *C*_*i*_=*c*_*i*_) of the *l*th joint from the *i*th patient is Markov and time homogeneous (although the marginal process is not assumed to be Markov and/or time homogeneous), the conditional likelihood contribution from the *l*th joint of the *i*th patient is

$$\prod_{j=2}^{m_{i}-1}P\left\{X_{i}^{l}\left(t_{ij+1}\right)=s_{ij+1}^{l}|X_{i}^{l}\left(t_{ij}\right)=s_{ij}^{l};z_{ij}^{l},u_{ij},v_{ij},c_{i}\right\}$$

where $s_{ij}^{l}$ represents the state corresponding to the specific combination of $\left(\{\overset{¯}{A},A\},\{\overset{¯}{D},D\}\right)$ observed at *t*_*ij*_ for the *l*th joint of the *i*th patient. More details on the likelihood construction for time homogeneous Markov models, particularly the form of the transition probabilities, can be found in Kalbfleisch and Lawless (1985). Appendix A provides the closed form transition probabilities of the six‐state process. Here, for simplicity, the likelihood contribution from the process between *t*_*i*1_ and *t*_*i*2_ is excluded because AMA cannot be calculated at *t*_*i*1_; it requires previous observations. As a diagnostic check of this simplification, we used activity at baseline to proxy AMA at baseline and this produced comparable results. If the assumption of independence between joints from the same patient is reasonable, conditional on the random effects *U*_*ij*_ and *V*_*ij*_, then

$$\begin{array}{cc} & L_{i}\left(\Theta |z_{ij},C_{i}\right)\\ & =\int_{-\infty }^{\infty }\int_{-\infty }^{\infty }\prod_{l=1}^{28}\prod_{j=2}^{m_{i}-1}P\left\{X_{i}^{l}\left(t_{ij+1}\right)=s_{ij+1}^{l}|X_{i}^{l}\left(t_{ij}\right)=s_{ij}^{l};z_{ij}^{l},u_{ij},v_{ij},c_{i}\right\}\phi \left(u_{ij},v_{ij};0,\Sigma \right)du_{ij}dv_{ij} \end{array}$$

represents the likelihood contribution from the *i*th patient, still conditional on the dynamic covariates and *C*_*i*_=*c*_*i*_. Here **Θ** is a vector containing all the unknown parameters to be estimated (baseline intensities, regression coefficients and random‐effects variance–covariance components) apart from the mover–stayer probabilities *π*_*i*_, *ϕ*(*u*_*ij*_,*v*_*ij*_;**0**,Σ) denotes the zero‐mean bivariate normal density with covariance matrix Σ and $z_{ij}=\left\{z_{ij}^{1},\ldots ,z_{ij}^{28}\right\}$. The overall marginal likelihood contribution from the *i*th patient is then

$$L_{i}\left(\Theta^{*}|z_{ij}\right)={\left\{\left(1-\pi_{i}\right)L_{i}\left(\Theta |z_{ij},C_{i}=1\right)\right\}}^{c_{i}^{*}}{\left\{\left(1-\pi_{i}\right)L_{i}\left(\Theta |z_{ij},C_{i}=1\right)+\pi_{i}L_{i}\left(\Theta |z_{ij},C_{i}=0\right)\right\}}^{1-c_{i}^{*}}$$

with the overall marginal likelihood *L*(**Θ** ^*^|**z**_*ij*_) obtained by taking the product of all likelihood contributions *L*_*i*_(**Θ** ^*^|**z**_*ij*_) from each patient. Here **Θ** ^*^={**Θ**,*π*_*i*_} and $c_{i}^{*}$ is a binary indicator such that $c_{i}^{*}=1$ if damaged joints are observed from patient *i* at their last clinic visit and $c_{i}^{*}=0$ otherwise. The bivariate numerical integrations were computed by firstly factorizing the bivariate density function into conditional densities, i.e. $\phi \left\{v_{ij};\rho u_{ij}\sigma_{v}/\sigma_{u},\sigma_{v}^{2}\left(1-\rho^{2}\right)\right\}\phi \left(u_{ij};0,\sigma_{u}^{2}\right)$, where $\sigma_{v}^{2}$ and $\sigma_{u}^{2}$ denote the respective variance components and *ρ* the correlation parameter, then using Gauss–Hermite quadrature to evaluate each integral with respect to *u*_*ij*_ and *v*_*ij*_ separately. The numbers of quadrature points for each integration were chosen to be 15 and 30 for the observation and patient level random‐effects models respectively. Weights and nodes from the quadrature rule were then calculated using the R (R Core Team, 2015) package statmod (Smyth *et al*., 2004). A sensitivity analysis indicated that further quadrature points provided negligible influence on parameter estimates and log‐likelihood values. The log‐likelihood was maximized using the BFGS (Broyden, 1970) optimization routine and asymptotic standard errors for parameter estimates were obtained by evaluating and then inverting the numerically derived Hessian matrix at the maximum likelihood estimates. The same estimation procedure was used in Yiu *et al*. (2017) to fit models that share the same essential characteristics with the models proposed in this paper. In their work, it was shown through simulation studies that, with a correctly specified model and sufficient follow‐up information, the estimation procedure in this paper can provide reliable estimates of their model parameters.

The next subsection provides results of fitting the proposed model to the data described in Section 2.

### 3.2 Results

In addition to the aforementioned covariates, adjustment covariates for type of joint, presence of opposite or contralateral joint damage (the same joint in the opposite hand is damaged; opposite joint damaged equals 1 and 0 otherwise), sex (male≡1 and female≡0), age at onset of arthritis (in years) and duration of arthritis (in years) are provided through $z_{ij}^{l}$. Joint type is represented through a five‐level categorical variable with levels metacarpophalangeal, proximal interphalangeal, distal interphalangeal, thumb metacarpophalangeal and baseline thumb proximal interphalangeal. This covariate was included in the transition intensities that were associated with $\overset{¯}{A}\to A$ and $\overset{¯}{D}\to D$ with preliminary analysis demonstrating little evidence of differential recovery rates from activity (i.e. $A\to \overset{¯}{A}$). The binary variable specifying the presence of opposite joint damage is motivated by previous analyses (Cresswell and Farewell, 2011; O’Keeffe *et al*., 2011) which indicate evidence of symmetric joint damage; the propensity of damage for a joint in a specific location to become damaged is increased if the contralateral joint in the other hand is earlier damaged.

Table 1 presents the results from fitting the model proposed (with dynamic covariates and an observation level random‐effects structure) to the 743 psoriatic arthritis patients described in Section 2. For comparative purposes, a model with patient level random effects, i.e. *U*_*ij*_=*U*_*i*_ and *V*_*ij*_=*V*_*i*_, was also fitted. The results of this model and a comparison with the results of the model proposed are provided in Appendix B. The larger log‐likelihood value of −47 389.11 for the model proposed compared with the log‐likelihood value of −52 784.84 for the comparative model would suggest a preference for the model proposed.

**Table 1:** Parameter estimates and 95% Wald intervals resulting from fitting the six‐state model (described in Section 3) to 743 psoriatic arthritis patients

*Parameter*	*Estimates for the following transitions:*		
	$\overset{¯}{A}\to A$	$A\to \overset{¯}{A}$	$\overset{¯}{D}\to D$
Damaged joint	−0.13 (−0.27, 0.0079)	−0.2 (−0.31, −0.088)	
Opposite joint damaged	0.17 (0.027, 0.3)	0.09 (−0.035, 0.21)	0.83 (0.6, 1.07)
Attained number of	0.033 (0.021, 0.045)	−0.0039 (−0.014, 0.0061)	0.22 (0.18, 0.25)
damaged joints			
Active joint			1.62 (1.3, 1.94)
AMA	2.72 (2.58, 2.87)	−0.49 (−0.61, −0.37)	2.01 (1.68, 2.34)
Metacarpophalangeal	0.3 (0.22, 0.372)		−0.84 (−1.10, −0.58)
Proximal interphalangeal	0.46 (0.38, 0.53)		−0.15 (−0.38, 0.089)
Distal interphalangeal	−0.18 (−0.26, −0.095)		0.49 (0.26, 0.73)
Thumb	0.45 (0.36, 0.55)		0.45 (0.17, 0.72)
metacarpophalangeal			
Sex	−0.69 (−0.79, −0.59)	0.017 (−0.055, 0.088)	0.2 (−0.047, 0.44)
Age at arthritis onset	0.0012 (−0.0031, 0.0055)	0.008 (0.0049, 0.011)	0.013 (0.0038, 0.023)
Arthritis duration	−0.021 (−0.027, −0.015)	0.0066 (0.0026, 0.011)	−0.01 (−0.023, 0.0028)
Stayer	1.99 (1.86, 2.12)	0.22 (0.11, 0.33)	
log(*λ*_0_)	−3.18 (−3.4, −2.95)	0.79 (0.63, 0.94)	−9.48 (−10.08, −8.89)
$\sigma_{u}^{2}$	2.07 (1.93, 2.21)		
*α*		−0.38 (−0.42, −0.35)	
$\sigma_{v}^{2}$			6.62 (5.89, 7.45)
*ρ*	0.16 (0.1, 0.21)		
*π*	0.14 (0.11, 0.18)		
Log‐likelihood	−47389.11		

#### 3.2.1 Damage process

From Table 1, it is clear that both opposite joint damage and the number of damaged joints are strongly and positively associated with an increased damage progression rate. Thus this analysis supports the results in O’Keeffe *et al*. (2011) concerning symmetry even after adjusting for a greater number of process features, although not adjusting for the stochastic nature of the opposite joint damage process. Activity, both current (the joint is active while adjusting for its stochastic nature) and history (as described by AMA), is seen to be strongly and positively associated with damage progression. Regarding inference, the confidence interval for the regression coefficient that is associated with current activity is narrower than the corresponding interval that was reported in O’Keeffe *et al*. (2011). This has probably resulted from using updated data and a more efficient estimation procedure (through dynamic covariates and random effects as opposed to a working independence assumption with a robust covariance matrix adjustment). These results therefore provide greater confidence in the strong positive association between activity and damage, and implicitly strengthens the argument that was made regarding causality.

#### 3.2.2 Activity process

There is evidence that the transition intensities that are associated with entering and leaving the active joint state reduces once a joint has become damaged. However, as the respective (95% Wald intervals) confidence intervals contain or are close to zero, this observation must currently be regarded as suggestive. The presence of opposite joint damage and the number of damaged joints seem to be moderately or weakly associated with the activity transition intensities. In particular little association is seen with transitioning from the active joint state to the not‐active joint state. The strong association between history of activity and current activity transition intensities is reassuring, since the interpretation of greater amounts of previous activity increasing the transition intensity to the active state while decreasing the transition intensity to the not‐active state is intuitive.

#### 3.2.3 Movers and stayers

The percentage of stayers (100*π*% where *π*=*π*_*i*_ ∀ *i*) was estimated to be 14% (11%, 18%). Empirically, when compared with the 69% of patients who did not develop any damage, this estimate may seem to be a considerable underestimate of the true stayer proportion. However, because of the relationship between activity and damage, it is conceivable that many of these patients (those who did not develop damaged joints) did not develop damage because they were in the not‐active state for long periods of continuous time, as opposed to being stayers *per se*. This observation is perhaps supported by Table 1, which suggests that movers have a vastly smaller transition intensity to the active state compared with stayers. A more specific investigation regarding the sojourn times of movers and stayers in the not‐active joint state follows in the next section. It is also worth noting that the transition intensity to the not‐active state is smaller for movers; however, it is far less pronounced.

**Figure 5:**
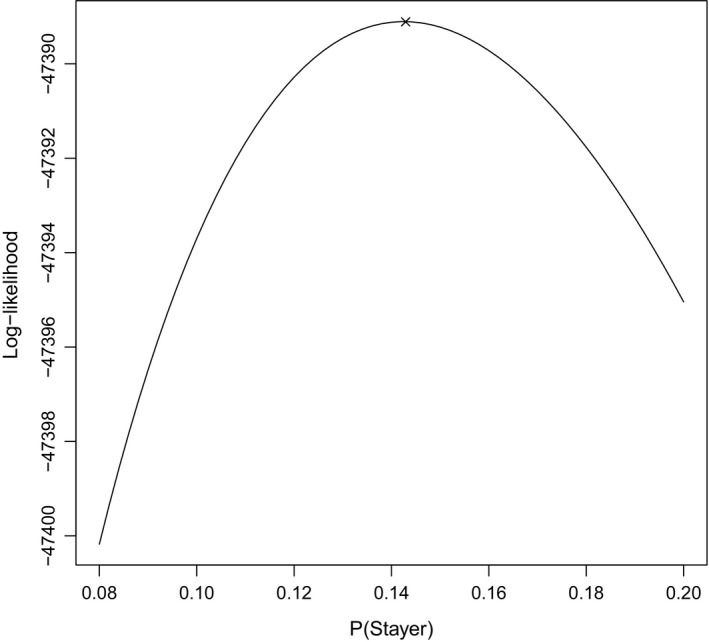
Plot of the profile log‐likelihood for *π* based on the six‐state model in Section 3 (×, point at which the numerical optimization procedure converged)

As a diagnostic check for parameter estimation, we plot the profile log‐likelihood for *π* in Fig. 5. From Fig. 5, it is clear that the numerical optimization routine converged at the maximum of the profile log‐likelihood for *π*. This indicates that the latent stayer proportion was identifiable under the assumptions of the six‐state model in Section 3 because the profile log‐likelihood has a quadratic shape.

## 4 Five‐state model for mean sojourn times

In many settings, clinical interest lies in understanding the mean sojourn times from a particular time point (the mean amount of time that a process will spend in a state from a particular time point before a transition occurs) as a function of covariates at that particular time point. When two or more transitions are possible from a state of interest, the current methodology of investigating a covariate effect involves fixing other covariates at specified values (usually at their means or as a description of a particular patient) and then calculating the difference in mean sojourn times from a particular time point for that state by varying the covariate of interest. This methodology is implicit because, under the current multistate modelling parameterization in terms of transition intensities, a direct interpretation of covariate effects on the mean sojourn times is not straightforward when two or more transitions are possible from the state of interest; the mean sojourn time is a non‐linear function of covariates from different transition intensities. This section considers the novel approach of modelling the mean sojourn times directly through a model reparameterization to obtain easily interpretable covariate effects on this quantity. In our situation, this approach is possible because there is a smooth bijection from the transition intensities to the mean sojourn times and jump probabilities (see the next paragraph). This implies that more elaborate but computationally intensive techniques such as the use of pseudo‐observations (Anderson and Perme, 2010) can be avoided.

**Figure 6:**
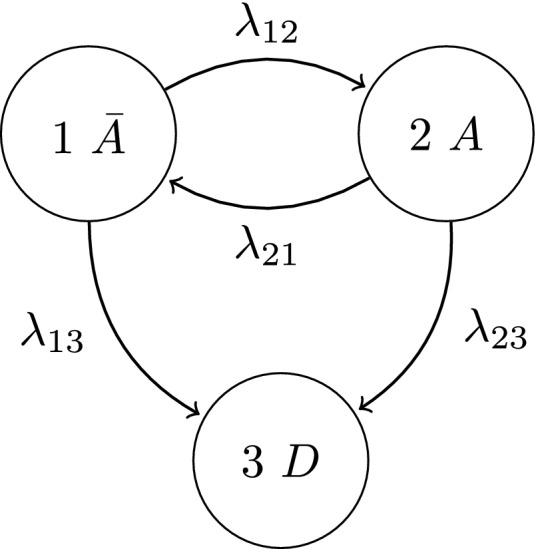
Multistate model describing activity and damage processes jointly for movers

**Figure 7:**
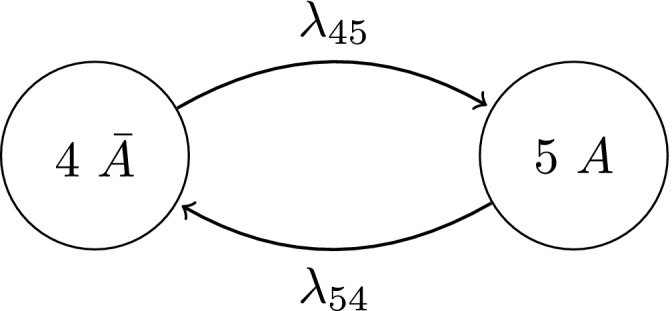
Multistate model describing the activity process for stayers

The specific context of interest concerns the sojourn times in the active and not‐active states before damage. For simplicity, we can therefore revert to a three‐state model for the movers by combining the activity process after damage has occurred into a single absorbing state, as depicted in Fig. 6. Similarly, the multistate diagram describing possible transitions for stayers is displayed in Fig. 7.

Although continuous time Markov processes can be viewed in terms of transition intensities specifying risks of transitioning through states (as thought of previously), another natural way to view such processes is in terms of sojourn times and jump probabilities being associated with each state. As mentioned, the sojourn time in a state from a particular time point describes the amount of time that the process will spend in that state from that particular time point before a transition occurs. Then, at the point of transitioning, the jump probabilities inform the multinomial distribution of the possible set of states in which the process will jump to next. For the conditional process that we consider (a continuous time Markov process), the sojourn time in state *r* from the time of entry into state *r* can be shown to have an exponential distribution with mean 1/Σ_*k*≠*r*_ *λ*_*rk*_ and jump probabilities given by $P\left(\text{jumps to state}\;k|\;\text{jumps from state}\;r\right)=\lambda_{rk}/\Sigma_{k\neq r}\lambda_{rk}$. See pages 259–260 in Grimmett and Stirzaker (2001) for more details. Thus, by using the lack of memory property of the exponential distribution, we make the simplifying assumption that the sojourn times from a particular time point do not depend on the (unobserved) time that has already elapsed in that state conditional on time‐dependent covariates and random effects at that particular time point and the time‐independent mover–stayer status.

To investigate the difference in the activity process between movers and stayers again more simply, we parameterize as follows:

$$\begin{array}{cc} \mu_{4ij}^{l} & =\mu_{1ij}^{l}\;\text{exp}\left(\beta_{\mathrm{Stayer}}^{\overset{¯}{A}}\right),\\ \mu_{5ij}^{l} & =\mu_{2ij}^{l}\;\text{exp}\left(\beta_{\mathrm{Stayer}}^{A}\right), \end{array}$$

where $\mu_{rij}^{l}$ represents the mean sojourn time in state *r* for the *l*th joint of the *i*th patient from time *t*_*ij*_. Regression models for the mean sojourn times and jump probabilities can then be specified as follows:

(3) $$\left.\begin{array}{cc} & \mu_{1ij}^{l}=\mu_{0}^{\overset{¯}{A}}\;\text{exp}\left(\beta^{\overset{¯}{A}}z_{ij}^{l}+u_{ij}\right),\\ & \mu_{2ij}^{l}=\mu_{0}^{A}\;\text{exp}\left(\beta^{A}z_{ij}^{l}+\alpha_{1}u_{ij}\right),\\ & p_{13ij}^{l}/\left(1-p_{13ij}^{l}\right)=p_{0}^{\overset{¯}{A}D}\;\text{exp}\left(\beta^{\overset{¯}{A}D}z_{ij}^{l}+v_{ij}\right),\\ & p_{23ij}^{l}/\left(1-p_{23ij}^{l}\right)=p_{0}^{AD}\;\text{exp}\left(\beta^{AD}z_{ij}^{l}+\alpha_{2}v_{ij}\right), \end{array}\right\}$$

where $p_{\overset{¯}{A}Dij}^{l}$ and $p_{ADij}^{l}$ denote the jump probabilities from state $\overset{¯}{A}\to D$ and *A*→*D* respectively for the *l*th joint of the *i*th patient at *t*_*ij*_. The right‐hand side of each regression equation in expression (3) contains a baseline (indicated by 0 in the subscript) multiplied by the exponent of the sum of a linear predictor and linear function of realizations of random effects, as before. Dynamic covariates (AMA and the attained number of damaged joints) and random effects are again included to reflect features of the processes that were described in Section 3. The random effects follow a zero‐mean bivariate normal distribution and are independent across time.

The model fitting procedure follows from Section 3.1 after having specified the transition probabilities which as a function of transition intensities can be found in Appendix C. Thus the following set of equations completes the procedure:

$$\begin{array}{cc} & \lambda_{12}=\left(1-p_{13}\right)/\mu_{1},\\ & \lambda_{13}=p_{13}/\mu_{1},\\ & \lambda_{21}=\left(1-p_{23}\right)/\mu_{2},\\ & \lambda_{23}=p_{23}/\mu_{2},\\ & \lambda_{45}=1/\mu_{4},\\ & \lambda_{54}=1/\mu_{5}. \end{array}$$

### 4.1 Results

**Figure 8:**
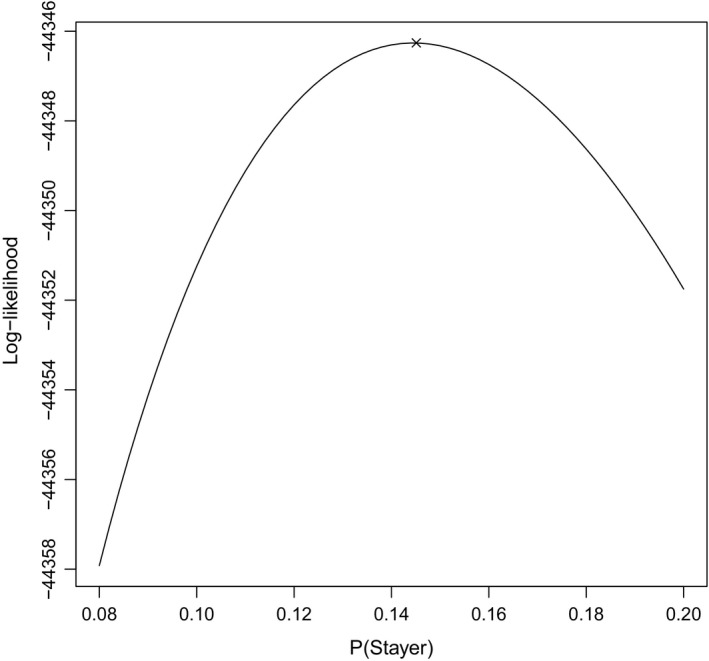
Plot of the profile log‐likelihood for *π* based on the five‐state model in Section 4 (×, point at which the numerical optimization procedure converged)

Along with dynamic covariates, the presence of opposite joint damage, sex, age at onset of arthritis and duration of arthritis were included in the analysis, as before. Table 2 presents the results from fitting the five‐state model that was described in Section 4 to the 743 psoriatic arthritis patients described in Section 2. From Table 2, the presence of opposite joint damage provides a slight increase in the mean sojourn time in the not‐active joint state and greatly increases the probability of directly transitioning to damage (as opposed to active and not damaged) once a transition occurs. However, when a joint is active, there is little evidence to suggest that opposite joint damage influences the sojourn times nor the next state probability. These results indicate that the presence of opposite joint damage is particularly relevant when a joint is not active. A large number of damaged joints, although substantially increasing the jump probabilities to damage as opposed to active or not active, provides little effect on the mean sojourn times in the active and not‐active states. As expected, greater amounts of previous activity (as described by AMA) decrease the mean sojourn time in the not‐active state and increase the mean sojourn time in the active state. Table 2 also suggests that AMA is strongly and positively associated with the jump probability to damage when in the active state but not in the not‐active state. Thus current activity is strengthened by the history of activity when dictating the next state of the process, but jumping to damage or active from the not‐active state could be unaffected by the history of activity. As hypothesized in the previous section, stayers have a far shorter mean sojourn time in the not‐active state, and a slightly shorter sojourn time in the active state. Finally, the estimated stayer proportion in Table 2 is approximately equivalent to the stayer proportion that was reported in Table 1. Under the assumptions of the five‐state model, the numerical optimization procedure could identify the parameter *π* as it converged at the maximum of its profile log‐likelihood (Fig. 8).

**Table 2:** Parameter estimates and 95% Wald intervals resulting from fitting the five‐state model (described in Section 4) to 743 psoriatic arthritis patients

*Parameter*	*Sojourn times*		*Jump probabilities*	
	$\overset{¯}{A}$	*A*	$\overset{¯}{A}\to D$	*A*→*D*
Opposite joint damaged	−0.28 (−0.44, −0.12)	−0.1 (−0.27, 0.061)	1.6 (0.87, 2.34)	0.54 (−0.18, 1.26)
Attained number of damaged joints	−0.09 (−0.11, −0.074)	0.0075 (−0.0057, 0.021)	0.21 (0.14, 0.29)	0.16 (0.11, 0.22)
AMA	−2.98 (−3.13, −2.83)	0.39 (0.26, 0.52)	−0.25 (−1.02, 0.52)	0.84 (0.21, 1.48)
Sex	0.68 (0.58, 0.78)	0.0053 (−0.067, 0.078)	1.24 (0.74, 1.74)	0.43 (−0.022, 0.89)
Age at arthritis onset	−0.0021 (−0.0064, 0.0023)	−0.0078 (−0.011, −0.0047)	1.24 (0.74, 1.74)	0.43 (−0.022, 0.89)
Arthritis duration	0.019 (0.014, 0.025)	−0.0036 (−0.0076, 0.00047)	0.037 (0.016, 0.059)	−0.023 (−0.051, 0.0053)
Stayer	−1.93 (−2.06, −1.8)	−0.17 (−0.29, −0.049)		
log(*μ*_0_)	3.04 (2.82, 3.25)	−0.83 (−0.99, −0.67)		
log(*p*_0_)			−9.27 (−10.9, −7.65)	−5.68 (−6.81, −4.55)
$\sigma_{u}^{2}$	2.05 (1.92, 2.2)			
*α*_1_		−0.35 (−0.39, −0.32)		
$\sigma_{v}^{2}$			17.4 (12.66, 23.92)	
*α*_2_				0.44 (0.32, 0.56)
*ρ*	0.0041 (−0.048, 0.056)			
*π*	0.15 (0.12, 0.18)			
Log‐likelihood	−44346.26			

Table 3 provides the results of fitting the five‐state model with a patient level random‐effects structure (i.e. *U*_*ij*_=*U*_*i*_ and *V*_*ij*_=*V*_*i*_ ∀ *j*) to the data that were described in Section 2. The resulting log‐likelihood value was −49 256.31, which is far smaller than −44 346.26 obtained from the model proposed. In this context, the observation level random‐effects structure, after including dynamic covariates, again seems to be the more appropriate random‐effects structure for the data.

**Table 3:** Parameter estimates and 95% Wald intervals resulting from fitting the five‐state model with a patient level random‐effects structure to 743 psoriatic arthritis patients

*Parameter*	*Sojourn times*		*Jump probabilities*	
	$\overset{¯}{A}$	*A*	$\overset{¯}{A}\to D$	*A*→*D*
Opposite joint damaged	−0.28 (−0.44, −0.11)	−0.19 (−0.35, −0.026)	0.95 (0.44, 1.46)	0.44 (0.01, 0.86)
Attained number of damaged joints	0.012 (−0.0022, 0.026)	−0.033 (−0.044, −0.022)	−0.3 (−0.45, −0.14)	0.056 (0.019, 0.093)
AMA	−1.98 (−2.11, −1.85)	0.29 (0.17, 0.4)	0.7 (0.068, 1.32)	0.87 (0.34, 1.4)
Sex	0.79 (0.7, 0.88)	0.012 (−0.054, 0.078)	1.14 (0.51, 1.77)	0.36 (−0.066, 0.79)
Age at arthritis onset	−0.014 (−0.019, −0.0098)	−0.0028 (−0.0054, −0.00025)	0.032 (0.0095, 0.054)	−0.0082 (−0.03, 0.013)
Arthritis duration	0.036 (0.032, 0.039)	−0.0024 (−0.0062, 0.0013)	0.18 (0.13, 0.23)	0.041 (0.017, 0.065)
Stayer	−2.23 (−2.36, −2.1)	−0.35 (−0.46, −0.23)		
log(*μ*_0_)	2.71 (2.49, 2.94)	−0.91 (−1.04, −0.77)		
log(*P*_0_)			−8.8 (−10.43, −7.18)	−5.61 (−6.59, −4.62)
$\sigma_{u}^{2}$	1.28 (1.16, 1.41)			
*α*_1_		−0.24 (−0.29, −0.19)		
$\sigma_{v}^{2}$			6.96 (4.41, 10.98)	
*α*_2_				0.63 (0.45, 0.81)
*ρ*	−0.057 (−0.15, 0.032)			
*π*	0.16 (0.13, 0.2)			
Log‐likelihood	−49256.31			

## 5 Discussion

This research was motivated by reproducing prior results with an updated data set and undertaking new investigations into disease course and progression. For this, a single unifying clustered multistate modelling framework which allows simultaneous investigations of multiple clinical aspects was proposed. The results obtained therefore yield greater confidence when compared with multiple univariate investigations, which were performed previously, since they are based on adjusting for other important process characteristics. From a clinical perspective, the relationship between activity and damage was demonstrated as pronounced since both history and current activity were positively related to damage progression and jumping to damage once a joint immediately leaves the active state. In terms of the reverse relationship, the onset of damage is seen to slow the activity process although the confidence intervals for the relevant regression coefficients indicate that no change is a distinct possibility, maybe because of far fewer observed transitions after damaged has occurred. Interestingly, both models seem to identify a subpopulation of approximately 15% who are rapidly fluctuating in their activity process yet are at minimal risk of damage, perhaps because they have shorter sojourn times in the active joint state. An avenue of future work could involve identifying these patients especially because their treatment strategies should conceivably not consist of potent drugs, which may cause unpleasant side effects, but soft drugs to reduce joint swelling and pain. It is also reassuring that neither model contradicts any well‐held clinical beliefs.

From a statistical point of view, the novel aspects of this research include the proposing of an observation level random‐effects structure combined with dynamic covariates, a mover–stayer structure whereby movers and stayers can have different effects on transition intensities in which they are not implicitly defined for and a model parameterization which allows easily interpretable covariate effects to act on the sojourn times and jump probabilities. In our context, the usefulness of the methodology proposed was demonstrated through new clinical insights and substantial improvements in likelihood values over the use of standard methodology (patient level random‐effect models). Although the methodology proposed was described in terms of specific, but fairly complex, six‐ and five‐state models for the margins, extensions to general clustered continuous time Markov models are straightforward. In particular, the proposed model parameterization in terms of sojourn times and jump probabilities, and mover–stayer effects on transition intensities are also applicable to univariate Markov multistate processes and therefore can provide a useful framework for inference in many clinical settings.

Overall, this research represents our efforts to provide a comprehensive investigation into many clinical aspects of interest at the finest level of detail (individual joint level). Although there are foreseeable model extensions, it is important to bear in mind the computationally intensive nature of fitting clustered multistate models with random effects, especially when reversible multistate models are involved (transitions to and from states exist). Some examples of potentially more appropriate extensions could include relaxing the time homogeneity assumption beyond adjusting for duration of arthritis, relating previous history to current transitions through more accurate measures than the proposed dynamic covariates, e.g. by specifying a flexible correlation structure for the observation level random effects, and dividing the damage onset state into various states of severity of damage. Such extensions, as with many others, will usually require transitioning from piecewise constant approximations to reflecting the true stochastic nature of the outcome, covariates and latent processes, which has been seen as one of the main drivers of making the model fitting procedure more complex, because of the larger number of integrations or differential equations that are required to be computed or solved. Nevertheless, as demonstrated here, it is important to identify and provide adjustments for important process characteristics where possible, in which model comparison is useful. With respect to understanding clinical aspects of psoriatic arthritis, this has provided new knowledge and greater confidence in prior results based on less general methodology.

## Appendix A

The transition probabilities, i.e. $p_{rs}\left(t\right)\equiv p_{rs}\left(t;\lambda \right)=P\left\{X\left(t+t_{0}\right)=s|X\left(t_{0}\right)=r;\lambda \right\}$ ∀ *t*_0_⩾0 where ***λ*** is a vector of the required transition intensities, for the four‐ and two‐state process depicted in Figs 2 and 3 respectively can be calculated by evaluating the matrix exponential of the relevant transition intensity matrix. Specifically, let

$$Q_{1}=\left(\begin{array}{cccc} -\lambda_{12}-\lambda_{13} & \lambda_{12} & \lambda_{13} & 0\\ \lambda_{21} & -\lambda_{21}-\lambda_{24} & 0 & \lambda_{24}\\ 0 & 0 & -\lambda_{34} & \lambda_{34}\\ 0 & 0 & \lambda_{43} & -\lambda_{43} \end{array}\right)\;\;;$$

then the transition probabilities for the four‐state process can be obtained from the (*r*,*s*)th entry of exp(*Q*_1_ *t*). We use Mathematica to compute the following matrix exponential and this results in

$$p_{11}\left(t\right)=\frac{\text{exp}(-\Lambda t/2)}{2\gamma }\left\{\left(\Lambda_{1}+\gamma \right)\;\text{exp}\left(-\gamma t/2\right)+\left(\gamma -\Lambda_{1}\right)\;\text{exp}\left(\gamma t/2\right)\right\},$$

$$p_{12}\left(t\right)=\frac{\lambda_{12}}{\gamma_{1}}\left[\text{exp}\left\{-\left(\Lambda -\gamma_{1}\right)t/2\right\}-\text{exp}\left\{-\left(\Lambda +\gamma_{1}\right)t/2\right\}\right],$$

$$\begin{array}{cc} p_{13}\left(t\right) & =\frac{\lambda_{43}}{\Lambda_{2}}+\frac{\text{exp}(-\Lambda_{2}t)}{\Lambda_{2}}\frac{\lambda_{13}\lambda_{34}\left(\lambda_{21}+\lambda_{24}-\Lambda_{2}\right)-\lambda_{12}\lambda_{24}\lambda_{43}}{\left(\lambda_{21}+\lambda_{24}-\Lambda_{2}\right)\left(\lambda_{13}-\Lambda_{2}\right)-\lambda_{12}\left(\lambda_{24}-\Lambda_{2}\right)}\\ & \;+\lambda_{13}\;\text{exp}\left\{-\left(\Lambda -\gamma \right)t/2\right\}\frac{\lambda_{12}\lambda_{21}+\left(\lambda_{12}+\lambda_{13}\right)\left(\Lambda_{1}-\gamma \right)/2+\lambda_{43}\left(-\Lambda_{1}+\gamma -2\lambda_{34}\right)/2+\gamma_{2}}{-{\left(\Lambda -\gamma \right)}^{3}/2+3{\left(\Lambda -\gamma \right)}^{2}\left(\Lambda +\Lambda_{2}\right)/4-\left(\Lambda -\gamma \right)\gamma_{3}+\gamma_{4}}\\ & \;+\lambda_{13}\;\text{exp}\left\{-\left(\Lambda +\gamma \right)t/2\right\}\frac{\lambda_{12}\lambda_{21}+\left(\lambda_{12}+\lambda_{13}\right)\left(\Lambda_{1}+\gamma \right)/2+\lambda_{43}\left(-\Lambda_{1}-\gamma -2\lambda_{34}\right)/2+\gamma_{2}}{-{\left(\Lambda +\gamma \right)}^{3}/2+3{\left(\Lambda +\gamma \right)}^{2}\left(\Lambda +\Lambda_{2}\right)/4-\left(\Lambda +\gamma \right)\gamma_{3}+\gamma_{4}}, \end{array}$$

$$p_{14}\left(t\right)=1-p_{11}\left(t\right)-p_{12}\left(t\right)-p_{13}\left(t\right),$$

where

$$\begin{array}{cc} & \Lambda =\lambda_{12}+\lambda_{13}+\lambda_{21}+\lambda_{24},\\ & \Lambda_{1}=\lambda_{12}+\lambda_{13}-\lambda_{21}-\lambda_{24},\\ & \Lambda_{2}=\lambda_{34}+\lambda_{43},\\ & \gamma =\surd \left[\Lambda^{2}-4\left\{\lambda_{12}\lambda_{24}+\lambda_{13}\left(\lambda_{21}+\lambda_{24}\right)\right\}\right],\\ & \gamma_{1}=\surd \left\{\lambda_{12}^{2}+2\lambda_{12}\left(\lambda_{13}+\lambda_{21}-\lambda_{24}\right)+{\left(-\lambda_{13}+\lambda_{21}+\lambda_{24}\right)}^{2}\right\},\\ & \gamma_{2}=\frac{\lambda_{43}}{\lambda_{13}}\left(\lambda_{12}\lambda_{24}+\lambda_{13}\lambda_{34}\right),\\ & \gamma_{3}=\Lambda_{2}\left(\lambda_{21}+\lambda_{24}\right)+\lambda_{13}\lambda_{21}+\left(\lambda_{12}+\lambda_{13}\right)\left(\lambda_{24}+\Lambda_{2}\right),\\ & \gamma_{4}=\Lambda_{2}\left\{\lambda_{13}\lambda_{21}+\lambda_{24}\left(\lambda_{12}+\lambda_{13}\right)\right\}. \end{array}$$

As the four‐state process is symmetric, it is easy to verify that

$$\begin{array}{cc} p_{21}\left(t;\lambda_{12},\lambda_{13},\lambda_{21},\lambda_{24},\lambda_{34},\lambda_{43}\right) & =p_{12}\left(t;\lambda_{21},\lambda_{24},\lambda_{12},\lambda_{13},\lambda_{43},\lambda_{34}\right),\\ p_{22}\left(t;\lambda_{12},\lambda_{13},\lambda_{21},\lambda_{24},\lambda_{34},\lambda_{43}\right) & =p_{11}\left(t;\lambda_{21},\lambda_{24},\lambda_{12},\lambda_{13},\lambda_{43},\lambda_{34}\right),\\ p_{23}\left(t;\lambda_{12},\lambda_{13},\lambda_{21},\lambda_{24},\lambda_{34},\lambda_{43}\right) & =p_{14}\left(t;\lambda_{21},\lambda_{24},\lambda_{12},\lambda_{13},\lambda_{43},\lambda_{34}\right),\\ p_{24}\left(t;\lambda_{12},\lambda_{13},\lambda_{21},\lambda_{24},\lambda_{34},\lambda_{43}\right) & =p_{13}\left(t;\lambda_{21},\lambda_{24},\lambda_{12},\lambda_{13},\lambda_{43},\lambda_{34}\right). \end{array}$$

Finally, we have

$$\begin{array}{cc} & p_{33}\left(t\right)=1-p_{34}\left(t\right),\\ & p_{34}\left(t\right)=\lambda_{34}\left\{1-\text{exp}\left(-\Lambda_{2}t\right)\right\}/\Lambda_{2},\\ & p_{43}\left(t\right)=\lambda_{43}\left\{1-\text{exp}\left(-\Lambda_{2}t\right)\right\}/\Lambda_{2},\\ & p_{44}\left(t\right)=1-p_{34}\left(t\right),\\ & p_{55}\left(t\right)=1-p_{56}\left(t\right),\\ & p_{56}\left(t\right)=\lambda_{56}\left\{1-\text{exp}\left(-\Lambda_{3}t\right)\right\}/\Lambda_{3},\\ & p_{65}\left(t\right)=\lambda_{65}\left\{1-\text{exp}\left(-\Lambda_{3}t\right)\right\}/\Lambda_{3},\\ & p_{66}\left(t\right)=1-p_{56}\left(t\right), \end{array}$$

where Λ_3_=*λ*_56_+*λ*_65_.

## Appendix B

Table 4 provides the results from fitting the six‐state model with a patient level random‐effects structure to the 743 psoriatic arthritis patients that was described in Section 2. Rather reassuringly, most regression coefficients of primary interest, including the stayer proportion estimate, from Table 4 are seen to have similar interpretations to those obtained from Table 1. The regression coefficients that are associated with the dynamic covariates AMA and attained number of damaged joints have, however, resulted in markedly different estimates from those seen in Table 1. Specifically, the effects of the dynamic covariates are greatly attenuated and, furthermore, the attained number of damaged joints is now seen to have a possibly counterintuitive negative association with damage progression. Both dynamic covariates and patient level random effects adjust for a patient's propensity to gain or recover from activity and to gain damage, and thus are likely to be confounded when introduced simultaneously in the model. The regression coefficients that are associated with the dynamic covariates are now perhaps more difficult to interpret than before. Aalen *et al*. (2008) provides a discussion on the relationship between dynamic models (with dynamic covariates) and frailty models (with patient level random effects).

**Table 4:** Parameter estimates and 95% Wald intervals resulting from fitting the six‐state model with a patient level random‐effects structure to 743 psoriatic arthritis patients

	*Results for the following transitions:*		
	$\overset{¯}{A}\to A$	$A\to \overset{¯}{A}$	$\overset{¯}{D}\to D$
Damaged joint	−0.046 (−0.18, 0.086)	−0.18 (−0.27, −0.069)	
Opposite joint damaged	0.13 (−0.0031, 0.27)	0.087 (−0.037, 0.21)	0.72 (0.52, 0.92)
Attained number of	−0.04 (−0.049, −0.03)	0.022 (0.013, 0.031)	−0.036 (−0.055, −0.018)
damaged joints			
Active joint			1.32 (1.03, 1.61)
AMA	1.8 (1.68, 1.93)	−0.34 (−0.45, −0.23)	1.7 (1.39, 2)
Metacarpophalangeal	0.23 (0.16, 0.3)		−0.81 (−1.1, −0.57)
Proximal interphalangeal	0.36 (0.29, 0.43)		−0.18 (−0.4, 0.049)
Distal interphalangeal	−0.19 (−0.27, −0.12)		0.36 (0.14, 0.58)
Thumb	0.39 (0.3, 0.48)		0.36 (0.095, 0.62)
metacarpophalangeal			
Sex	−0.73 (−0.8, −0.66)	0.011 (−0.051, 0.073)	−0.2 (−0.45, 0.042)
Age at arthritis onset	0.014 (0.011, 0.018)	0.0042 (0.0015, 0.0069)	−0.012 (−0.024, −0.00034)
Arthritis duration	−0.037 (−0.04, −0.033)	0.0058 (0.0022, 0.0095)	0.057 (0.047, 0.067)
Stayer	2.25 (2.12, 2.37)	0.37 (0.26, 0.47)	
log(*λ*_0_)	−2.88 (−3.07, −2.69)	0.8 (0.66, 0.94)	−7 (−7.55, −6.45)
$\sigma_{u}^{2}$	1.33 (1.21, 1.46)		
*α*		−0.23 (−0.28, −0.19)	
$\sigma_{v}^{2}$			3.19 (2.73, 3.73)
*ρ*	0.28 (0.2, 0.35)		
*π*	0.17 (0.13, 0.21)		
Log‐likelihood	−52784.84		

## Appendix C

The transition probabilities of the three‐state model were again calculated using the matrix exponential function in Mathematica. This resulted in

$$\begin{array}{cc} & p_{11}\left(t\right)=\frac{x_{1}\;\text{exp}\left(r_{1}t\right)-x_{2}\;\text{exp}\left(r_{2}t\right)}{x_{1}-x_{2}},\\ & p_{12}\left(t\right)=\frac{x_{1}x_{2}\left\{\text{exp}\left(r_{2}t\right)-\text{exp}\left(r_{1}t\right)\right\}}{x_{1}-x_{2}},\\ & p_{13}\left(t\right)=1-p_{11}\left(t\right)-p_{12}\left(t\right),\\ & p_{21}\left(t\right)=\frac{\text{exp}\left(r_{1}t\right)-\text{exp}\left(r_{2}t\right)}{x_{1}-x_{2}},\\ & p_{22}\left(t\right)=\frac{x_{1}\;\text{exp}\left(r_{2}t\right)-x_{2}\;\text{exp}\left(r_{1}t\right)}{x_{1}-x_{2}},\\ & p_{23}\left(t\right)=1-p_{21}\left(t\right)-p_{22}\left(t\right),\\ & p_{3j}\left(t\right)=1_{j=3}, \end{array}$$

where **1**_*j*=3_ is an indicator function taking the value 1 when *j*=3 and 0 otherwise, and

$$\begin{array}{cc} & r_{1}=\frac{\lambda_{11}+\lambda_{22}+\surd \left\{{\left(\lambda_{11}-\lambda_{22}\right)}^{2}+4\lambda_{12}\lambda_{21}\right\}}{2},\\ & r_{2}=\frac{\lambda_{11}+\lambda_{22}-\surd \left\{{\left(\lambda_{11}-\lambda_{22}\right)}^{2}+4\lambda_{12}\lambda_{21}\right\}}{2},\\ & x_{j}=\frac{r_{j}-\lambda_{22}}{\lambda_{21}}. \end{array}$$

Here *λ*_11_=−*λ*_12_−*λ*_13_ and *λ*_22_=−*λ*_21_−*λ*_23_.
